# Supplementary material for: Chromosome-scale genome assembly of the European common cuttlefish Sepia officinalis
Source: eLife. 2026 May 28;14:RP107393. doi: 10.7554/eLife.107393 (PMC13218726; doi:10.7554/eLife.107393)
Supplement: MDAR checklist [file elife-107393-mdarchecklist1.docx]

**Materials Design Analysis Reporting (MDAR)**

**Checklist for Authors**

The [MDAR framework](https://osf.io/xfpn4/) establishes a minimum set of requirements in transparent reporting mainly applicable to studies in the life sciences.

*eLife* asks authors to **provide detailed information within their article** to facilitate the interpretation and replication of their work. Authors can also upload supporting materials to comply with relevant reporting guidelines for health-related research (see [EQUATOR Network](http://www.equator-network.org/%20)), life science research (see the [BioSharing Information Resource](http://biosharing.org/)), or animal research (see the [ARRIVE Guidelines](http://www.plosbiology.org/article/info:doi/10.1371/journal.pbio.1000412) and the [STRANGE Framework](https://doi.org/10.1038/d41586-020-01751-5); for details, see *eLife*’s [Journal Policies](https://reviewer.elifesciences.org/author-guide/journal-policies)). Where applicable, authors should refer to any relevant reporting standards materials in this form.

For all that apply, please note **where in the article** the information is provided. Please note that we also collect information about data availability and ethics in the submission form.

**Materials:**

| **Newly created materials** | **Indicate where provided: section/figure legend** | **N/A** |
| --- | --- | --- |
| The manuscript includes a dedicated "materials availability statement" providing transparent disclosure about availability of newly created materials including details on how materials can be accessed and describing any restrictions on access. | Materials and Methods, "Data availability" section. Genome assembly and raw sequencing data are deposited under NCBI BioProject PRJNA1091451 and SRA study SRP570862; genome assembly is available on NCBI (GCA_050097725.1); annotation files are available at https://public.brain.mpg.de/Laurent/sepoff/annotation/ and doi:10.17617/3.CGO7QG. |  |
|  |  |  |
| **Antibodies** | **Indicate where provided: section/figure legend** | **N/A** |
| For commercial reagents, provide supplier name, catalogue number and [RRID](https://scicrunch.org/resources), if available. | N/A — no antibodies were used in this study. | X |
|  |  |  |
| **DNA and RNA sequences** | **Indicate where provided: section/figure legend** | **N/A** |
| Short novel DNA or RNA including primers, probes: Sequences should be included or deposited in a public repository. | N/A — no novel short DNA or RNA sequences were generated in this study.  The four qPCR primers used for sex genotyping (SepOff_chr2_auto_G2_F/R targeting an autosomal control locus on chromosome 2; SepOff_chr46_sex_H2_F/R targeting a sex-chromosomal locus on chromosome 46) were taken from the cited source publication (preprint doi:10.1101/2025.10.28.685099) and were not designed in this study; they were validated against the S. officinalis assembly generated here. Their sequences are nevertheless reproduced in Materials and Methods ("Sex genotyping by qPCR") and in the accompanying Key Resources Table for the reader's convenience. |  |
|  |  |  |
| **Cell materials** | **Indicate where provided: section/figure legend** | **N/A** |
| Cell lines: Provide species information, strain. Provide accession number in repository OR supplier name, catalog number, clone number, OR RRID. | N/A — no cell lines were used. | X |
| Primary cultures: Provide species, strain, sex of origin, genetic modification status. | N/A — no primary cultures were used. | X |
|  |  |  |
| **Experimental animals** | **Indicate where provided: section/figure legend** | **N/A** |
| Laboratory animals or Model organisms: Provide species, strain, sex, age, genetic modification status. Provide accession number in repository OR supplier name, catalog number, clone number, OR RRID. | Materials and Methods, "Animal husbandry" and "Tissue preparation" sections.  Species: Sepia officinalis (European cuttlefish), wild-type (no genetic modification).  Individual 1 — used for PacBio HiFi long-read DNA, Iso-Seq, Omni-C, and short-read Illumina DNA libraries: 6-month-old adult, F1 hatched in captivity from eggs obtained from the commercial supplier Flying Sharks – consultoria e inovação, Lda. (Rua Farrobim do Sul 116, 9900-361 Horta, Azores, Portugal; info@flyingsharks.eu); sex genotyped as male by qPCR (see Laboratory protocol row).  Individual 2 — used for short-read Illumina RNA-seq: 8-month-old adult, F0 hatched in captivity from eggs collected in Normandie, France (provided by Université de Caen Normandie, France); sex not determined. |  |
| Animal observed in or captured from the field: Provide species, sex, and age where possible. | N/A — both individuals were laboratory-reared in captivity from the egg stage. No animals were observed in or captured directly from the field by the authors. | X |
|  |  |  |
| **Plants and microbes** | **Indicate where provided: section/figure legend** | **N/A** |
| Plants: provide species and strain, ecotype and cultivar where relevant, unique accession number if available, and source (including location for collected wild specimens). | N/A — no plants were used as experimental subjects. | X |
| Microbes: provide species and strain, unique accession number if available, and source. | N/A — no microbes were used in this study. | X |
|  |  |  |
| **Human research participants** | **Indicate where provided: section/figure legend) or state if these demographics were not collected** | **N/A** |
| If collected and within the bounds of privacy constraints report on age, sex, gender and ethnicity for all study participants. | N/A — no human research participants. | X |

**Design:**

| **Study protocol** | **Indicate where provided: section/figure legend** | **N/A** |
| --- | --- | --- |
| If the study protocol has been pre-registered, provide DOI. For clinical trials, provide the trial registration number OR cite DOI. | N/A — descriptive genome assembly and annotation study; no pre-registered protocol or clinical trial. | X |
|  |  |  |
| **Laboratory protocol** | **Indicate where provided: section/figure legend** | **N/A** |
| Provide DOI OR other citation details if detailed step-by-step protocols are available. | Materials and Methods.  Iso-Seq full-length cDNA protocol: described in detail in Cartolano et al. 2016 (PLOS ONE 11:e0157779, doi:10.1371/journal.pone.0157779).  Sex genotyping by tissue gDNA extraction and qPCR: methodology adapted from Rubino et al., 2025 (doi:10.1101/2025.10.28.685099). Only the genomic DNA extraction (from tissue rather than from skin swabs) and qPCR portions of the source protocol were performed. |  |
|  |  |  |
| **Experimental study design (statistics details) *** | | |
| **For in vivo studies: State whether and how the following have been done** | **Indicate where provided: section/figure legend. If it could have been done, but was not, write “not done”** | **N/A** |
| Sample size determination | N/A — single-individual reference genome assembly and annotation; sample size is the conventional design for a reference-quality genome and is not amenable to formal power calculation. | X |
| Randomisation | N/A — descriptive genome project; no experimental groups or treatment allocation. | X |
| Blinding | N/A — descriptive genome project; no experimental groups or treatment allocation. | X |
| Inclusion/exclusion criteria | N/A at the animal level (single individuals used per modality). Data-level filtering criteria for downstream analyses (e.g., RNA-seq gene-count filter of ≥10 counts in ≥3 samples; GO terms restricted to those annotating 5–500 expressed genes) are stated in Materials and Methods, "Bulk RNA-seq analysis". |  |
|  |  |  |
| **Sample definition and in-laboratory replication** | **Indicate where provided: section/figure legend** | **N/A** |
| State number of times the experiment was replicated in the laboratory. | Materials and Methods (multiple sub-sections).  A single biological individual was used per sequencing modality, with no biological replication of the genome assembly itself. Long-read DNA: one library, sequenced across 5 PacBio SMRT cells. Iso-Seq: one pooled library across 8 tissues sequenced on 1 SMRT cell, plus a separate optic-lobe library on a 2nd SMRT cell. Omni-C: one library. Short-read DNA: one library. Short-read RNA-seq: separate libraries prepared from multiple tissues (different brain areas, skin, retina) of a single individual, sequenced twice on NextSeq500 and NextSeq2000. |  |
| Define whether data describe technical or biological replicates. | Materials and Methods, "Short-read RNA library preparation and sequencing" and "Bulk RNA-seq analysis".  The short-read RNA-seq data contain technical replicates at two levels:  1. Two libraries were prepared from each tissue (different brain regions, skin, retina, mantle/epidermis, arm/tentacle) of the single F0 individual.  2. Each library was sequenced twice, once on an Illumina NextSeq500 and once on an Illumina NextSeq2000, providing two technical replicates per library, and four replicates per tissue (except for the retina, which were only sequenced once on the NextSeq500).  Reads from all replicates were combined prior to gene-level counting and differential expression analysis on a tissue level. No technical replicates were performed for any other sequencing modality (long-read DNA, Iso-Seq, Omni-C, short-read DNA). |  |
|  |  |  |
| **Ethics** | **Indicate where provided: section/submission form** | **N/A** |
| Studies involving human participants: State details of authority granting ethics approval (IRB or equivalent committee(s), provide reference number for approval. | N/A — no human participants. | X |
| Studies involving experimental animals: State details of authority granting ethics approval (IRB or equivalent committee(s), provide reference number for approval. | Materials and Methods, "Animal husbandry" and "Tissue preparation" sections.  Procedures complied with EU Directive 2010/63/EU, the German animal welfare act, and FELASA guidelines, and followed the Guidelines for the Care and Welfare of Cephalopods in Research (Fiorito et al. 2015; Ponte et al. 2023) and the cephalopod anaesthesia/euthanasia recommendations of Andrews et al. 2013. Institutional project licence number: F126/1025. The terminal anaesthesia carried out for tissue collection is not classified as a regulated procedure under EU Directive 2010/63/EU. |  |
| Studies involving specimen and field samples: State if relevant permits obtained, provide details of authority approving study; if none were required, explain why. | Materials and Methods, "Animal husbandry" section.  Cuttlefish eggs were obtained from the licensed commercial supplier Flying Sharks – consultoria e inovação, Lda. (Rua Farrobim do Sul 116, 9900-361 Horta, Azores, Portugal), and the Université de Caen Normandie,  (UMR 6552 EthoS, Ethologie Animale et Humaine CNRS-Rennes I-Caen, Neuro-Ethologie Cognitive des Céphalopodes (NECC), Campus 5, J. Horowitz,  14032 Caen, France) which hold the relevant collection authorisations. An independent field-collection permit was therefore not required by the authors. |  |
|  |  |  |
| **Dual Use Research of Concern (DURC)** | **Indicate where provided: section/submission form** | **N/A** |
| If study is subject to dual use research of concern regulations, state the authority granting approval and reference number for the regulatory approval. | N/A — study describes a non-pathogen invertebrate reference genome and is not subject to dual-use research of concern regulations. | X |

**Analysis:**

| **Attrition** | **Indicate where provided: section/figure legend** | **N/A** |
| --- | --- | --- |
| Describe whether exclusion criteria were pre-established. Report if sample or data points were omitted from analysis. If yes, report if this was due to attrition or intentional exclusion and provide justification. | Materials and Methods.  All exclusion / inclusion criteria were pre-established and are stated in the relevant analysis sections.  (i) Bulk RNA-seq: genes retained only if ≥10 counts in ≥3 samples prior to DESeq2 ("Bulk RNA-seq analysis"); GO terms restricted to those annotating 5–500 genes in the expressed-gene universe.  (ii) Coverage analysis: uniquely mapping reads only (STAR); MAPQ ≥ 10 for HiFi count_coverage; only chromosomes with ≥10% reduction in median normalised depth considered for sex-chromosome calls.  (iii) Synteny analysis: minimum block size 5 anchor genes, maximum 5 intervening non-anchors, no secondary syntenic hits, etc. (parameter values in "Whole genome alignment and synteny analysis").  (iv) Gene-family analysis: orthogroups present in a single species were excluded; orthogroups with ≥100 copies in any species were separated prior to CAFE5 modelling ("Gene family expansion analysis").  No animal-level exclusions occurred; the F1 individual was the sole source of all DNA, Iso-Seq, Omni-C, and short-read DNA data. |  |
|  |  |  |
| **Statistics** | **Indicate where provided: section/figure legend** | **N/A** |
| Describe statistical tests used and justify choice of tests. | Materials and Methods (multiple sub-sections).  Coverage analysis: empirical p-values for breakpoint trans-contact rates against the genome-wide background distribution; one-tailed Wilcoxon rank-sum test for the three DToL breakpoints jointly.  Sex-chromosome analysis: one-sided Wilcoxon rank-sum test of per-chromosome normalised depth windows against all remaining chromosomes; p-values Benjamini-Hochberg adjusted.  Gene-family expansion: CAFE5 v5.1.1 — four candidate models compared (base; Gamma with k = 2, 3, 4 rate categories); the Gamma k = 3 model was selected on the basis of the best (lowest) final log-likelihood; significant families called at p < 0.05 after Bonferroni correction.  RNA-seq differential expression: DESeq2 v1.42.0 Wald test in a one-vs-all design; log2 fold changes shrunk with apeglm; tissue markers defined by padj < 0.05 (Benjamini-Hochberg), log2FC > 1, mean target-tissue expression > 5.  GO enrichment: hypergeometric over-representation test via clusterProfiler's enricher(); Benjamini-Hochberg adjustment; FDR < 0.05. |  |
|  |  |  |
| **Data availability** | **Indicate where provided: section/submission form** | **N/A** |
| For newly created and reused datasets, the manuscript includes a data availability statement that provides details for access (or notes restrictions on access). | Materials and Methods, "Data availability" section. |  |
| When newly created datasets are publicly available, provide accession number in repository OR DOI and licensing details where available. | Materials and Methods, "Data availability" section.  Genome assembly and raw data: NCBI BioProject PRJNA1091451; SRA study SRP570862. Genome assembly GCA_050097725.1. Annotation files: https://public.brain.mpg.de/Laurent/sepoff/annotation/ and doi:10.17617/3.CGO7QG. |  |
| If reused data is publicly available provide accession number in repository OR DOI, OR URL, OR citation. | Main text, Table 2 ("Overview of gene annotation of 13 molluscan species used for gene family analysis"). Accession numbers and source URLs are given there for each reused proteome / genome (RefSeq, Ensembl, GWH, and custom sources). |  |
|  |  |  |
| **Code availability** | **Indicate where provided: section/figure legend** | **N/A** |
| For any computer code/software/mathematical algorithms essential for replicating the main findings of the study, whether newly generated or re-used, the manuscript includes a data availability statement that provides details for access or notes restrictions. | Materials and Methods, "Data availability" section. |  |
| Where newly generated code is publicly available, provide accession number in repository, OR DOI OR URL and licensing details where available. State any restrictions on code availability or accessibility. | Materials and Methods, "Data availability" section.  Code for genome assembly and annotation: https://gitlab.mpcdf.mpg.de/mpibr/laur/cuttlefishomics/soffgenome under MIT license. |  |
| If reused code is publicly available provide accession number in repository OR DOI OR URL, OR citation. | Materials and Methods. All reused software is cited inline with tool name, version, and reference (e.g., hifiasm, YAHS, BRAKER3, RepeatMasker, RepeatModeler, GENESPACE, OrthoFinder, MCScanX, CAFE5, STAR, DESeq2, clusterProfiler, etc.). See also the accompanying Key Resources Table for full citations, DOIs and (where available) RRIDs. |  |

**Reporting:**

The MDAR framework recommends adoption of discipline-specific guidelines, established and endorsed through community initiatives.

| **Adherence to community standards** | **Indicate where provided: section/figure legend** | **N/A** |
| --- | --- | --- |
| State if relevant guidelines (e.g., ICMJE, MIBBI, ARRIVE, STRANGE) have been followed, and whether a checklist (e.g., CONSORT, PRISMA, ARRIVE) is provided with the manuscript. | Materials and Methods, "Animal husbandry" and "Tissue preparation" sections.  Animal work followed: EU Directive 2010/63/EU; the German animal welfare act; FELASA guidelines; the Guidelines for the Care and Welfare of Cephalopods in Research (Fiorito et al. 2015; Ponte et al. 2023); and the cephalopod anaesthesia/euthanasia recommendations of Andrews et al. 2013.  ARRIVE: a separate ARRIVE checklist is not provided as animal use was limited to tissue collection under terminal anaesthesia (non-regulated procedure under EU Directive 2010/63/EU); the items relevant to such use are covered in this MDAR checklist.  STRANGE: N/A — no behavioural component. |  |

* We provide the following guidance regarding transparent reporting and statistics; we also refer authors to [Ten common statistical mistakes to watch out for when writing or reviewing a manuscript](https://doi.org/10.7554/eLife.48175).

**Sample-size estimation**

- You should state whether an appropriate sample size was computed when the study was being designed
- You should state the statistical method of sample size computation and any required assumptions
- If no explicit power analysis was used, you should describe how you decided what sample (replicate) size (number) to use

**Replicates**

- You should report how often each experiment was performed
- You should include a definition of biological versus technical replication
- The data obtained should be provided and sufficient information should be provided to indicate the number of independent biological and/or technical replicates
- If you encountered any outliers, you should describe how these were handled
- Criteria for exclusion/inclusion of data should be clearly stated
- High-throughput sequence data should be uploaded before submission, with a private link for reviewers provided (these are available from both GEO and ArrayExpress)

**Statistical reporting**

- Statistical analysis methods should be described and justified
- Raw data should be presented in figures whenever informative to do so (typically when N per group is less than 10)
- For each experiment, you should identify the statistical tests used, exact values of N, definitions of center, methods of multiple test correction, and dispersion and precision measures (e.g., mean, median, SD, SEM, confidence intervals; and, for the major substantive results, a measure of effect size (e.g., Pearson's r, Cohen's d)
- Report exact p-values wherever possible alongside the summary statistics and 95% confidence intervals. These should be reported for all key questions and not only when the p-value is less than 0.05.

**Group allocation**

- Indicate how samples were allocated into experimental groups (in the case of clinical studies, please specify allocation to treatment method); if randomization was used, please also state if restricted randomization was applied
- Indicate if masking was used during group allocation, data collection and/or data analysis
